# DBSCAN-SWA: An Integrated Tool for Rapid Prophage Detection and Annotation

**DOI:** 10.3389/fgene.2022.885048

**Published:** 2022-04-19

**Authors:** Rui Gan, FengXia Zhou, Yu Si, Han Yang, Chuangeng Chen, Chunyan Ren, Jiqiu Wu, Fan Zhang

**Affiliations:** ^1^ HIT Center for Life Sciences, School of Life Science and Technology, Harbin Institute of Technology, Harbin, China; ^2^ Department of Hematology/Oncology, Boston Children’s Hospital, Harvard Medical School, Boston, MA, United States; ^3^ APC Microbiome Ireland, School of Microbiology, University College Cork, Cork, Ireland

**Keywords:** prophage, phage, density-based spatial clustering, sliding window, phage-host interaction

## Abstract

As an intracellular form of a bacteriophage in the bacterial host genome, a prophage usually integrates into bacterial DNA with high specificity and contributes to horizontal gene transfer (HGT). With the exponentially increasing number of microbial sequences uncovered in genomic or metagenomics studies, there is a massive demand for a tool that is capable of fast and accurate identification of prophages. Here, we introduce DBSCAN-SWA, a command line software tool developed to predict prophage regions in bacterial genomes. DBSCAN-SWA runs faster than any previous tools. Importantly, it has great detection power based on analysis using 184 manually curated prophages, with a recall of 85% compared with Phage_Finder (63%), VirSorter (74%), and PHASTER (82%) for (Multi-) FASTA sequences. Moreover, DBSCAN-SWA outperforms the existing standalone prophage prediction tools for high-throughput sequencing data based on the analysis of 19,989 contigs of 400 bacterial genomes collected from Human Microbiome Project (HMP) project. DBSCAN-SWA also provides user-friendly result visualizations including a circular prophage viewer and interactive DataTables. DBSCAN-SWA is implemented in Python3 and is available under an open source GPLv2 license from https://github.com/HIT-ImmunologyLab/DBSCAN-SWA/.

## Introduction

Bacteriophages are viruses that specifically infect their bacterial hosts. Passive replication of the phage genome relies on integration into the host’s chromosome and becoming a prophage (Panis et al., 2010). Nearly half of the sequenced bacteria are lysogens, representing a tremendous and previously under-explored source of phages. Phages coexist and evolve with bacteria, influencing the entire ecological environment. Recently, phage therapy, defined as using phages to treat bacterial infections, has also been greatly emphasized. Therefore, the identification of prophages in their host genomes is critical not only for understanding their biological mechanisms but also for developing therapeutic strategies.

Several computational tools have been developed to predict putative prophage regions. Phage_Finder (Fouts, 2006) is a standalone software based on a heuristic algorithm to identify prophage regions in completely sequenced bacterial genomes. VirSorter (Roux et al., 2015) is a tool to detect viral segments in microbiome sequencing data. PHASTER is a popular webserver to identify and annotate prophage sequences in prokaryotic genomes and plasmids (Arndt et al., 2016). Prophage Hunter (Song et al., 2019) is a one-stop webserver to identify prophage regions in bacterial genomes and evaluate the activity of the prophages. All these tools have substantially revolutionized the prediction of prophages in bacterial genomes. However, PHASTER and Prophage Hunter only support predictions using the webserver but cannot perform large-scale predictions for high-throughput microbiome sequencing data. Though supporting prophage detection from massive bacterial genomes, Phage_Finder and VirSorter have limitations in speed and predictive power. To accommodate running speed, detection rate and accuracy, and data scale, we introduce DBSCAN-SWA, a tool to detect prophages in a high-throughput mode, which outperforms previous tools in running time and detection efficiency (Table 1). DBSCAN-SWA can be run either as a web server (http://www.microbiome-bigdata.com/PHISDetector/index/tools/DBSCAN-SWA) or as a command line tool available at https://github.com/HIT-ImmunologyLab/DBSCAN-SWA/.

**TABLE 1:** Performance comparison of DBSCAN-SWA with other prophage detection tools on *Xylella fastidiosa* Temecula1genome sequence (NC_004556).

	DBSCAN-SWA	Prophage hunter	PHASTER	Phage_Finder	VirSorter
Last updated	2020	2019	2016	2006	2015
Input type	FASTA/GBK	FASTA	FASTA/GBK	Special format	FASTA
Timing	∼1.5 min	∼9 min	Slow (queuing)	∼2 min	∼15 min
Standalone	YES	NO	NO	YES	YES
Interactive	YES	YES	YES	NO	YES
Att site prediction	YES	YES	YES	NO	NO
Gene annotation	YES	YES	NO	YES	NO
Recall	100%	N/A	∼71%	∼57%	∼57%

N/A means more tests are needed. Timing was tested on a Linux platform for *Xylella fastidiosa* Temecula1, which has a genome of approximately 2.5 Mbp. Slow means depending on the queuing time. No in “standalone” means only a webserver is provided. Recall was calculated for *Xylella fastidiosa* Temecula1 using (Multi−) FASTA, sequences. Special input files are needed for Phage_Finder including pep/.ffa, .ptt, and .con/.fna files.

## Materials and Methods

### Prophage Detection

**FIGURE 1:**
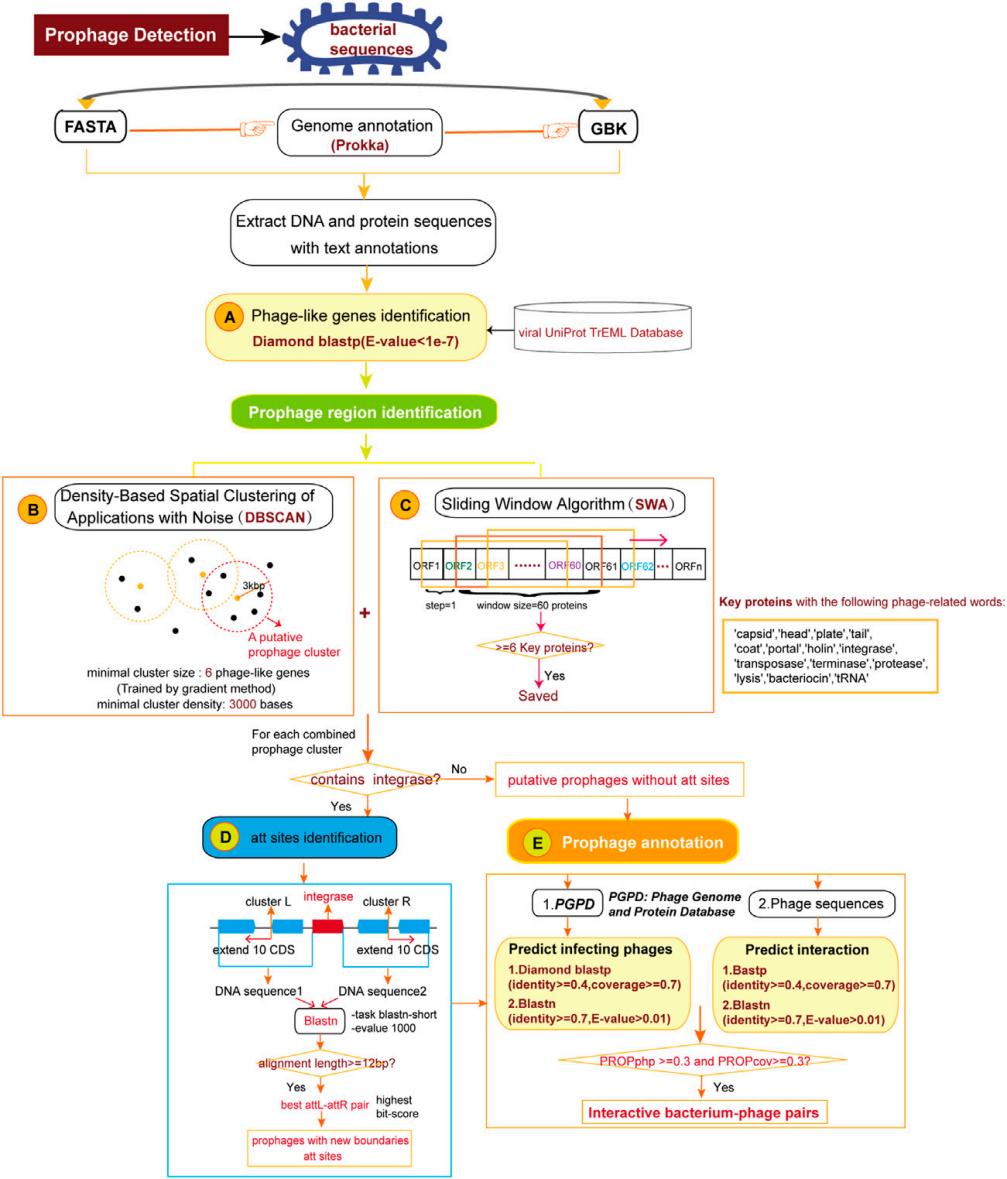
The pipeline for detection and annotation of prophages for bacterial genomes. **(A)** Identification of phage or phage-like proteins. **(B)** Detection of prophage clusters by Density-Based Spatial Clustering of Application with Noise algorithm. **(C)** Detection of prophage clusters by Sliding Window Algorithm. **(D)** Identification of attachment sites in prophage clusters. **(E)** Annotation of infecting phages for the predicted prophage regions.

Prophage regions are composed of phage or phage-like genes clustered in bacterial genome (Zhou et al., 2011). DBSCAN-SWA implements an algorithm combining density-based spatial clustering of applications with noise (DBSCAN) and a sliding window algorithm (SWA) to detect putative prophage sequences on bacterial genomes referring to the algorithm principle underlying PHASTER (Figure 1). Prokka (Seemann, 2014) is a command line software tool to fully, accurately and fast annotate a draft bacterial genome. If a multi-FASTA input file is received, gene prediction and annotation will be performed by Prokka (Seemann, 2014) to obtain a standard GenBank format file with tRNA sites additionally annotated using ARAGORN (Laslett and Canback, 2004). If a GenBank annotated file is submitted, gene annotations including protein sequences, functional descriptions, and tRNA sites will be extracted for subsequent analysis. First, Phage or phage-like proteins are identified using Diamond BLASTP (Buchfink et al., 2015) to search against DBSCAN-SWA’s local viral UniProt TrEML reference database (Consortium, 2013). Proteins with BLASTP e-values less than 1e-7 are considered as phage-like genes. Second, the positions of the hit proteins are used to detect minimal prophage clusters by DBSCAN with the default parameters of minimal cluster size set as 6 proteins and minimal cluster density set as 3,000 bases. These two parameters are the minimal number of phage-like genes required to form a prophage cluster (set to 6 proteins as the default parameter) and the maximal spatial distance between two neighbor genes within the same cluster, which reflects the protein density within the prophage region (set to 3,000 bps as the default parameter). These two parameters are learned using a gradient method based on 184 manually curated prophage regions (Casjens, 2003) by trying the minimal prophage size from 6 to 10 proteins (step = 1) and the protein density from 3,000 to 10000 bp (step = 1,000 bp). Third, in parallel, DBSCAN-SWA uses a sliding window based strategy (SWA) to search for putative prophage regions. Each window contains 60 annotated proteins and is used to search for phage-related proteins with specific keywords, such as “protease” and “integrase”. The windows with at least 6 phage-related proteins are retained and the minimal sub-region containing all detected phage-related proteins are returned as potential minimal prophage clusters (Figure 1C). Fourth, DBSCAN-SWA merges the minimal clusters, detected either by DBSCAN or SWA, that have intersections. Fifth, DBSCAN-SWA identifies putative attachment sites (att) in those merged clusters containing “integrase”, because integrase enzyme encoded within temperate phages typically determines the integration site specificity (Williams, 2002). Using the integrase protein as an anchor, the sequences of 10 upstream and downstream proteins on the bacterial genome are extracted to detect the putative attL-attR pairs using BLASTN with the parameters “-task blastn-short –evalue 1,000”. The attL-attR pair with the highest bit score and length >= 12 bp is considered as the putative att sites (Figure 1D). Finally, each prophage region is assigned a taxonomy by a majority vote based on the taxonomic information of all phage-like genes detected within the region.

### Prophage Annotation

DBSCAN-SWA provides two ways to annotate infecting phages for the predicted prophage regions. If candidate phage genomic sequence(s) in multi-FASTA format is given, DBSCAN-SWA will perform homologous protein alignment by BLASTP and nucleotide alignment by BLASTN to evaluate the similarity between the integrated prophage(s) and the phage genome(s) based on three prophage-related features proposed in PHISDetector (Zhou et al., 2022) (Supplementary Table S1). Alternatively, users can predict the infecting phages by a Diamond BLASTP and a BLASTN search against our local custom phage genome and protein database (PGPD) (Figure 1E), which contains 10,463 complete phage genome sequences and 684,292 nonredundant phage proteins collected from millardlab (http://millardlab.org/bioinformatics/bacteriophage-genomes/).

## Results and Discussion

### Overview of DBSCAN-SWA

**FIGURE 2:**
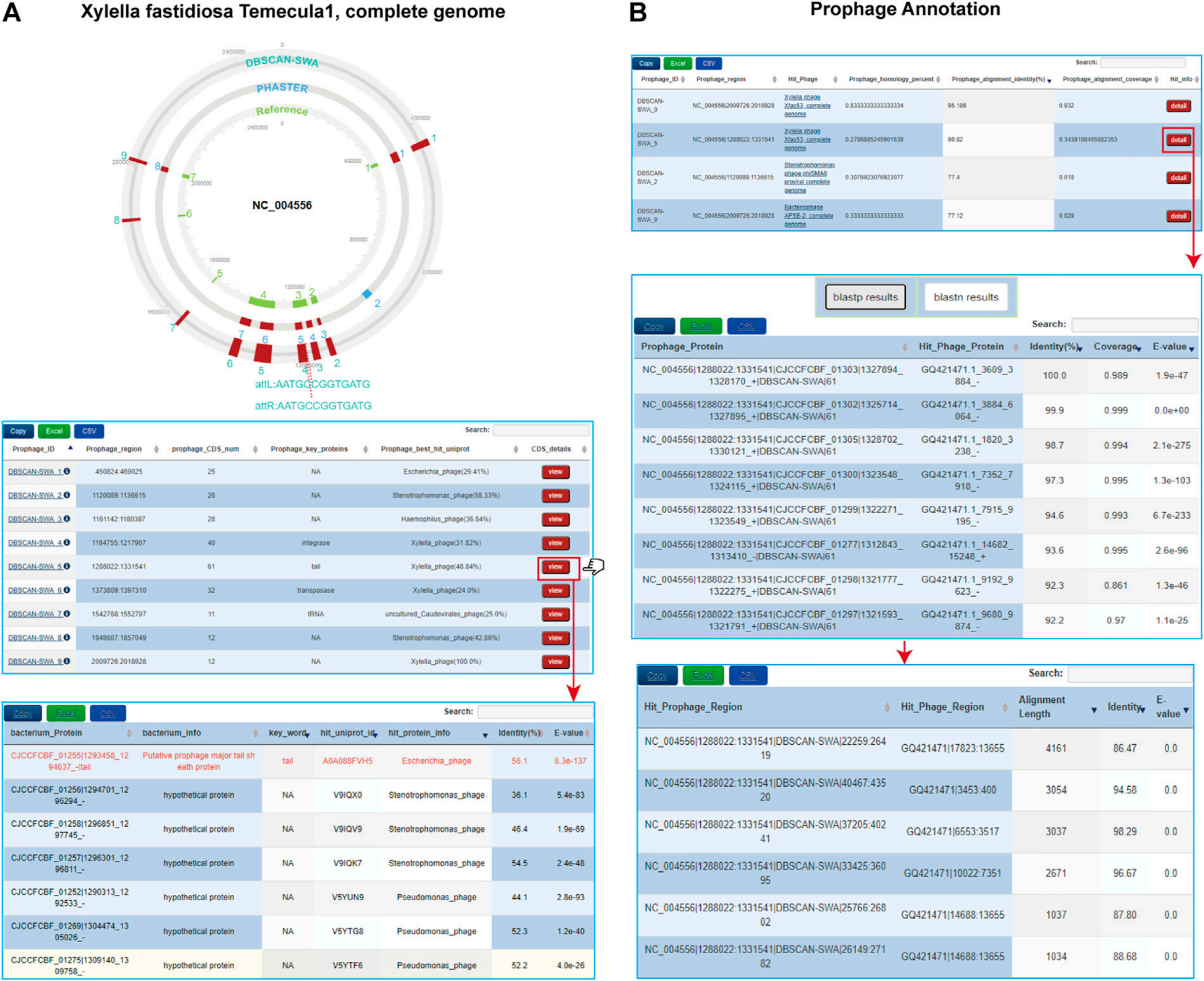
Visualizations of DBSCAN-SWA for prophage detection. **(A)** Interactive XHTML visualization of predicted prophages for Xylella fastidiosa Temecula1 (NC_004556) including a circular prophage viewer to display colored prophage regions with att sequences and interactive tables to display the detailed information of each prophage. **(B)** Interactive tables to display the predicted infecting phages and hit information for Xylella fastidiosa Temecula1 by using the parameter: “--add annotation PGPD”.

With the growing bacterial next-generation sequencing (NGS) data, there is a massive demand for a tool that is capable of detecting prophage regions in a high-throughput mode. DBSCAN-SWA was developed in order to achieve fast and accurate identification of prophage sequences from bacterial genomes. DBSCAN-SWA is an integrated tool for the detection of prophages that combines ORF prediction and gene function annotation, phage-like gene clusters detection, attachment site identification, and infecting phage annotation (Figure 1), with well-designed result visualizations and data tables (Figure 2). Currently, VirSorter and Phage_Finder, are the only two standalone software for prophage detection that are suitable for high-throughput sequencing data analysis. DBSCAN-SWA outperforms these tools in installation and usage (Figure 2A; Table 1). DBSCAN-SWA obtained the recall and precision of both 100% on *Xylella fastidiosa* Temecula1 genome sequence (NC_004556). VirSorter is difficult to install due to its complex configuration environment, and Phage_Finder requires special input files including pep/.ffa, .ptt, and .con/.fna files and only fits for complete sequence analysis. In contrast, DBSCAN-SWA is well packaged, easy to install and use, and supports analysis for both completely sequenced genomes and incompletely assembled contigs in multiple FASTA or GBK file format. Therefore, it is especially convenient for high-throughput metagenomics sequencing data analysis. Meanwhile, DBSCAN-SWA provides prophage annotation for the detected prophages using a custom phage database and evaluates the interaction of the bacterial genomes and infecting phages based on three prophage-related features. As a standalone software, DBSCAN-SWA also provides a user-friendly interactive HTML result page for users to browse the predicted prophages in a genome viewer and detailed prophage information and bacterium-phage interactions in data tables (Figures 2A, B). Furthermore, DBSCAN-SWA enables users to adjust the parameters for phage-like protein identification, att site identification and prophage annotation to meet their requirements for achieving proper prediction results based on their knowledge of prophages and phage-host interactions.

### The Advantages of DBSCAN-SWA Over PHASTER

DBSCAN-SWA implements an algorithm combining DBSCAN and SWA to detect the putative prophages, with reference to PHASTER which is the most popular prophage detection tool but without standalone version or available source code. Moreover, we made improvements from several aspects, including more comprehensive input processing, enhanced phage-like protein detection efficiency, flexible parameter setting, and att sites identification. First, we enable DBSCAN-SWA to accept two types of bacterial sequence files (multi-FASTA and GenBank format) as input. Second, we greatly improved the efficiency of DBSCAN-SWA compared with tools such as PHASTER, by an initial search for phage or phage-like proteins against DBSCAN-SWA’s local viral UniProt TrEML reference database using Diamond BLASTP (e-values < 1e-7) (Buchfink et al., 2015), this will speed up by orders of magnitude. Third, considering distinct biological features of prophages from different bacterial species, DBSCAN-SWA allows users to flexibly modify the two key parameters of DBSCAN to detect phage protein clusters, while PHASTER provides only fixed parameters. Forth, DBSCAN-SWA predict user-defined bacterium-phage interactions through prophage signal by calculating three prophage-related features (Supplementary Table S1) while PHASTER only predicted infecting phages from their local database. Fifth, DBSCAN-SWA provides HTML feature with the best of both worlds (command line and visualization) while PHASTER only supported visualizations by web server for users.

### The Performance of DBSCAN-SWA Compared With Other Methods

**FIGURE 3:**
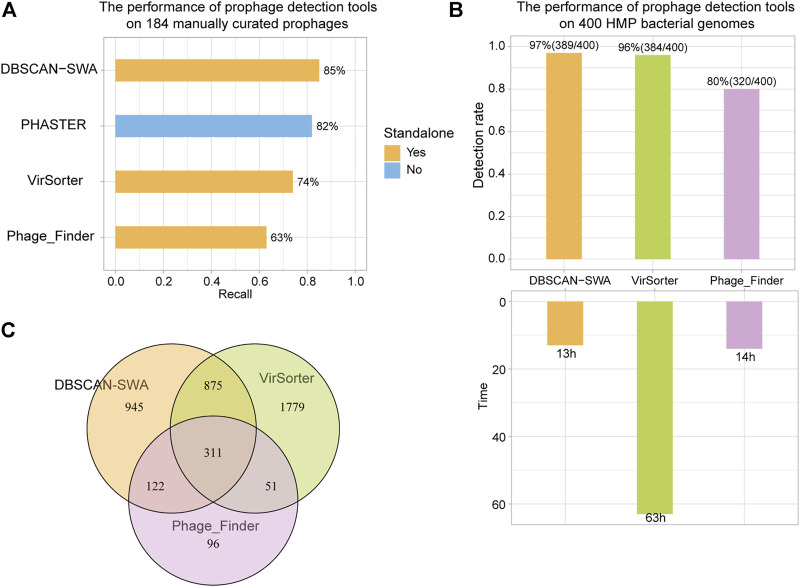
Recall of prophage detection tools for 184 manually curated prophages and 400 HMP bacterial genomes. **(A)** Recall of detection results for 184 manually curated prophages using DBSCAN-SWA, PHASTER, VirSorter, and Phage_Finder. **(B)** Detection rate and time of predicting prophages for 400 HMP bacterial genomes. **(C)** Shared prophages of DBSCAN-SWA, Phage_Finder, and VirSorter for 400 HMP bacterial genomes.

To evaluate the performance of DBSCAN-SWA, 184 manually curated prophages from 50 completely sequenced bacterial genomes were collected to examine the prophage prediction capability based on recall (the percentage of correctly predicted prophages from 184 curated prophages) and precision (the number of correctly predicted prophages divided by the total number of predicted prophages). The results showed that DBSCAN-SWA performed the best with recalls of 85% for (Multi-) FASTA sequence input, compared to PHASTER, Phage_Finder and VirSorter with recall of 82, 63, and 74%, respectively (Figure 3A; Supplementary Table S2). Moreover, DBSCAN-SWA presented better predictive power in NGS data than Phage_Finder and VirSorter based on the analysis of 19,989 contigs of 400 bacterial genomes (∼1 GB) in human gastrointestinal tract collected from HMP (https://www.hmpdacc.org/hmp). DBSCAN-SWA was able to predict 2,253 prophages on 1,469 contigs from 389 bacterial genomes in approximately 13 h with a detection rate (the percentage of bacterial genomes with putative prophages detected) of 97% (389/400), while Phage_Finder predicted 580 prophages from 261 bacterial genomes in approximately 14 h with a detection rate of 65% (261/400). Compared to VirSorter, DBSCAN-SWA runs 6 times faster, by taking approximately 63 h to predict 3,016 prophages from 384 (384/400 = 96%) bacterial genomes (Figure 3B; Supplementary Tables S3–S5). Meanwhile, DBSCAN-SWA also has a high degree of agreement with the prediction results of Phage_Finder, sharing 433 prophages (433/580 = 74.7%), and a lower degree with VirSorter with 1,186 shared prophage regions (1,186/3,016 = 39.3%). Nevertheless, VirSorter only shares 362 prophages with Phage_Finder (362/580 = 62.4% and 362/3,016 = 12%) (Figure 3C). All the results above prove that DBSCAN-SWA can predict putative prophages for high-throughput sequencing data and outperforms existing prophage prediction tools in terms of efficiency and predictability (Table 1; Supplementary Table S2)**.** DBSCAN-SWA can be further improved in several aspects. First, since identification of phage like proteins is the key step to predict putative prophages, we could further enhance the detection of phage or phage-like proteins by searching against a more complete viral database or using the hidden Markov model-based probabilistic algorithm (Cimermancic et al., 2014) to identify more novel phage-like protein families. Second, applying suitable clustering algorithm could greatly improve the detection of phage-like gene clusters, and subsequently influence the accuracy of detecting putative prophage regions. As DBSCAN and SWA are traditional unsupervised clustering algorithms, we propose that combining other algorithms specific for prophage detection or similar biological problems may improve the identification for novel prophage regions. Third, we will continue to improve DBSCAN-SWA by incorporating continuous efforts on identifying and evaluating active prophages to contribute to the study of phage physiology and co-evolution between phage and bacteria (Ofir and Sorek, 2018). We expect our work will inspire more researchers to combine both computational prediction and experimental validation to a broader range of studies including prophage inactivation.

## Conclusion

Nearly half of the sequenced bacteria are lysogens, representing a tremendous and previously under-explored source of prophages. Our study developed a novel software suitable for high-throughput prophage detection. It outperforms previous prophage detection tools in both running time and detection efficiency, and will extremely promote prophage detection for exponentially increasing microbial genomic sequences, especially for metagenomics sequencing.

## Data Availability

Publicly available datasets were analyzed in this study. This data can be found here: http://www.microbiome-bigdata.com/PHISDetector/static/download/DBSCAN-SWA/db.tar.gz.
